# Open MoA: revealing the mechanism of action (MoA) based on network topology and hierarchy

**DOI:** 10.1093/bioinformatics/btad666

**Published:** 2023-10-31

**Authors:** Xinmeng Liao, Mehmet Ozcan, Mengnan Shi, Woonghee Kim, Han Jin, Xiangyu Li, Hasan Turkez, Adnane Achour, Mathias Uhlén, Adil Mardinoglu, Cheng Zhang

**Affiliations:** Department of Protein Science, Science for Life Laboratory, KTH-Royal Institute of Technology, 17121 Stockholm, Sweden; Department of Protein Science, Science for Life Laboratory, KTH-Royal Institute of Technology, 17121 Stockholm, Sweden; Department of Medical Biochemistry, Faculty of Medicine, Zonguldak Bulent Ecevit University, 67630 Zonguldak, Turkey; Department of Protein Science, Science for Life Laboratory, KTH-Royal Institute of Technology, 17121 Stockholm, Sweden; Department of Protein Science, Science for Life Laboratory, KTH-Royal Institute of Technology, 17121 Stockholm, Sweden; Department of Protein Science, Science for Life Laboratory, KTH-Royal Institute of Technology, 17121 Stockholm, Sweden; Guangzhou National Laboratory, Guangzhou, Guangdong Province 510005, China; Department of Medical Biology, Faculty of Medicine, Atatürk University, Erzurum 25240, Turkey; Science for Life Laboratory, Department of Medicine, Solna, Karolinska Institute, 17176 Stockholm, Sweden; Department of Protein Science, Science for Life Laboratory, KTH-Royal Institute of Technology, 17121 Stockholm, Sweden; Department of Protein Science, Science for Life Laboratory, KTH-Royal Institute of Technology, 17121 Stockholm, Sweden; Centre for Host-Microbiome Interactions, Faculty of Dentistry, Oral & Craniofacial Sciences, King’s College London, London SE1 9RT, United Kingdom; Department of Protein Science, Science for Life Laboratory, KTH-Royal Institute of Technology, 17121 Stockholm, Sweden

## Abstract

**Motivation:**

Many approaches in systems biology have been applied in drug repositioning due to the increased availability of the omics data and computational biology tools. Using a multi-omics integrated network, which contains information of various biological interactions, could offer a more comprehensive inspective and interpretation for the drug mechanism of action (MoA).

**Results:**

We developed a computational pipeline for dissecting the hidden MoAs of drugs (Open MoA). Our pipeline computes confidence scores to edges that represent connections between genes/proteins in the integrated network. The interactions showing the highest confidence score could indicate potential drug targets and infer the underlying molecular MoAs. Open MoA was also validated by testing some well-established targets. Additionally, we applied Open MoA to reveal the MoA of a repositioned drug (JNK-IN-5A) that modulates the PKLR expression in HepG2 cells and found STAT1 is the key transcription factor. Overall, Open MoA represents a first-generation tool that could be utilized for predicting the potential MoA of repurposed drugs and dissecting *de novo* targets for developing effective treatments.

**Availability and implementation:**

Source code is available at https://github.com/XinmengLiao/Open_MoA.

## 1 Introduction

Drug repurposing, especially computational drug repurposing, is a novel approach for exploring new medical uses of existing drugs or drug candidates (Parvathaneni *et al.* 2019). Compared to traditional drug development processes, drug repurposing offers great advantages in pharmaceutical research, including speeding up the research and lowering the risks (Pushpakom *et al.* 2019, Fetro and Scherman 2020). To date, various computational approaches including signature matching (Shukla *et al.* 2021), molecular docking (Singh *et al.* 2020), genetic association (Irham *et al.* 2020), gene co-expression network analysis (Li *et al.* 2022), and pathway mapping (Greene *et al.* 2015) have been developed for the systematic analysis of large-scale omics data, resulting in the identification of potential candidates and pave the way for discovering the underlying pathogenesis of a variety of diseases (Pushpakom *et al.* 2019). However, most of the drug repurposing methods are black boxes and the mechanism of action (MoA) of the repurposed drug are usually remain unknown.

Systems biology-based network analysis can assist in elucidating the global drug/protein relationships and MoA of the drugs. By depicting entities as nodes and relationships as edges, a comprehensive network enables the incorporation of multiple types of data, modeling their correlations, and generation of putative subnetworks. Integrated networks (INs) combine diverse levels of desired biological information into an intensive network with known or predicted relationship. INs could help to better interpret complicated biological associations and address new opportunities for drug repurposing. Several types of INs, such as drug–disease (Hu *et al.* 2020), drug–drug (Sadegh *et al.* 2021), drug–gene (Liang *et al.* 2023), and protein interactions INs (Muthaiyan *et al.* 2021), have been used to decipher the drug MoAs. However, the transcription factors (TFs) are often overlooked in these methods. Since TFs are acting important roles in physiological and pathological processes of human diseases (Jiramongkol and Lam 2020, Alharbi *et al.* 2021, Henley and Koehler 2021), it could also be the leading candidates of MoAs in drug developments. Hence, the discovery of key TFs and the potential intact reacting pathway could help with unveiling more specific and underlying MoAs.

In this study, we presented the Open MoA: for revealing the MoAs using IN. Essentially, the method identifies MoAs of targeted interventions (siRNA or small molecule drugs) by searching the interactions that are mostly likely activated within the context-specific IN. We demonstrated that Open MoA could reconstruct well-known mechanisms of TGF-b1, WNT1, and metformin. In addition, we revealed and experimentally validated the previously unknown MoA of a repurposed drug (JNK-IN-5A) for non-alcoholic fatty liver disease via Open MoA.

## 2 Materials and methods

**Figure 1.**
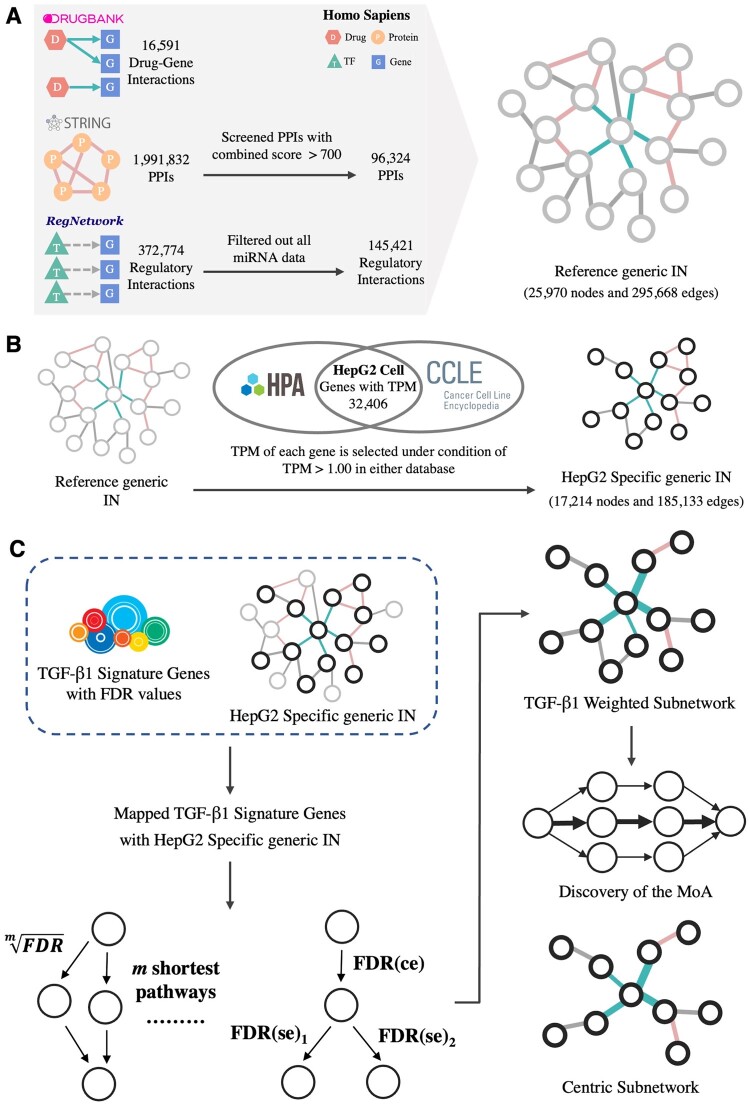
Schematic illustration of Open MoA. (A) Data management and integration. (B) Construction of the HepG2-specific IN. (C) Construction of the TGF-β1 weighted subnetwork and application of Open MoA to discover the fundamental MoA and identify the key targets in the TGF-β1 centric subnetwork.

We made three important assumptions in Open MoA: (i) the multi-omics generic IN is comprehensive and it includes all potential interactions in cells of *Homo sapiens*; (ii) all edges in the IN are regarded as independent biological events in the calculation; and (iii) the dysregulated genes are most likely to be modulated via the shortest path. The first assumption is grounded with existing knowledge of biological interactions, while the second and third ones are used by many previous studies to simplify the calculations and infer the functional molecules (Liu *et al.* 2016, Fu *et al.* 2017, Ren *et al.* 2018, Thilaga *et al.* 2018, Weiskittel *et al.* 2022). Figure 1 presents the Open MoA workflow and its application in constructing the TGF-β signaling pathway. In brief, using a given starting point (e.g. the drug-binding site), Open MoA traces all possible pathways to each gene that is perturbed by the drug treatments through the shortest path, particularly the differentially expressed genes (DEGs). Thereafter, the Programme calculates edge-specific confidence scores to establish the subnetwork(s) or pathway(s) that are most likely affected by each given starting point. Each of the steps will be explained in detail hereafter.

### 2.1 Construction of the reference IN

To construct a comprehensive reference IN for elucidating the underlying MoA of repurposed drugs, we first defined the entities (nodes) of the reference IN and relationships (edges) between them. Specifically, drug–targeted gene interactions, PPIs, and TF–gene regulatory interactions with experimental evidence were retrieved from DrugBank v5.1.9 (Wishart *et al.* 2018), STRING database v11.5 (Szklarczyk *et al.* 2017), and RegNetwork database (Liu *et al.* 2015), respectively. Only *H.sapiens* interactions from these three databases are kept where applicable. For PPIs from the STRING database, we only selected the ones with high confidence scores (combined score >700) (von Mering *et al.* 2005) whose experimental or experimental transferred scores is more than 0 to refine the network and denoise the less confident data. For TF–gene regulatory interactions, only the relationships between TFs and genes are remained from RegNetwork.

### 2.2 Construction of the HepG2-specific IN

All the interactions were merged to generate a directed reference IN with node information and interaction types. The direction of drugs-to-gene and TF-to-gene edges follows the original source, while PPI edges are set to bidirectional. In order to specify the reference IN to HepG2 cell line, gene transcriptomic data of HepG2 cell line from The Human Protein Atlas (Thul and Lindskog 2018) and the Cancer Cell Line Encyclopedia (Barretina *et al.* 2012) were used to filter nodes in the reference generic IN. Genes with low expression (TPM<1.00) in either dataset are filtered out, and the remaining genes were kept to construct a HepG2-specific IN (Fig. 1B).

### 2.3 Construction of the HepG2-specific weighted IN

In this step, we would like to assess the confidence scores by using the transcriptomic changes after perturbation. Essentially, we would like to find out how the perturbation of the starting point (either a drug or a target gene) would modulate the end points (the genes that changed their expression) through the HepG2-specific IN. In order to do so, the shortest path between the starting point and the endpoints are identified using igraph v1.3.5 in R. Notably, since gene expression is probably influenced by TFs, the last edge in every shortest path is set as the regulatory interaction that starts from a TF.

Next, we would like to calculate the probability of perturbation for each edge by the confidence scores from all shortest paths. Here, the false discovery rates (FDRs) of transcriptomic changes (e.g. differential expression) on each target gene (endpoint) were defined as the Penalty Score (PScore) of the endpoint. It could be interpreted as the probability that their expression has no change. Therefore, we could use this PScore as an indicator to estimate the chance of a perturbation existence. Based on this, we assigned weights step-by-step to all edges from the starting point (drug-binding site or gene modulation target).

In case of edges that involved in multiple paths going to different endpoints, we apply the fundamental statistical independency theory (Theodoridis 2015) and calculate their joint probability as the product of their own probabilities, which means:

(1) $$P\left(A,B\right)=P\left(A\right)\times P\left(B\right).$$

Therefore, we applied Equation (1) to calculate the proximity for each edge within drug actions. If there are totally *m* shortest topological pathways between a starting point and an endpoint of a drug action, the PScore of each single edge within a drug action will be assigned evenly with *n*th root of the FDR value, which is expressed as following:

(2) $$\mathrm{PScore}(\mathrm{se})\;=\sqrt[{m}]{\mathrm{PScore}\left(\mathrm{ep}\right),}$$

where PScore(ep), PScore(se), and *m* refer to the PScores of an endpoint, a single edge, and the total number of shortest topological pathways, respectively. The assumption here is that if the endpoint is perturbed, at least one of the pathways is activated.

An edge involved in more than one drug action is defined as a common edge. The PScore of a common edge will be the product of all the PScores it obtained according to Equation (1), which is expressed as following:

(3) $$\mathrm{PScore}\left(\mathrm{ce}\right)={\mathrm{PScore}\left(\mathrm{se}\right)}_{1}\times \;{\mathrm{PScore}\left(\mathrm{se}\right)}_{2}\ldots \;\times \mathrm{PScore}\left(\mathrm{se}\right)n,$$

where PScore(ce) represents the PScore of a common edge. PScore(se) is the PScore of a single edge computed by Equation (2). *n* represents the total number of drug actions that a common edge is involved.

Eventually, all edges in the HepG2-specific IN will be calculated via Equations (2) and (3). In this way, the unweighted IN is then transferred into a weighted subnetwork, with all edges having their own PScore [PScore(se) or PScore(ce)]. Therefore, the confidence score of each edge for assessing the interaction importance is expressed as following:

(4) Confidence Score=1-Penalty Score.

Using the perturbation-specific weighted network, the step-by-step MoA with the highest confidence score between the starting point and a certain endpoint could be identified by looking for the shortest topological pathway with the highest confidence score by igraph v v1.3.5 in R.

### 2.4 Comprehensive methodology and supplementary details

In-depth explanations concerning the methodologies adopted, including data collection processes, computational analysis, the development and utilization of the Open MoA script, and the experimental validation procedures are available in Supplementary Material S1.

## 3 Results

### 3.1 The Open MoA allows unambiguous reconstruction of the TGF-β signaling pathway

We developed Open MoA for discovering the underlying MoAs of the repurposed drugs. As shown in Fig. 1A, 16 591 drug–gene interactions were retrieved from the DrugBank database, containing 5806 drugs and 2773 target genes. For the PPIs, 1 755 256 of 1 991 832 interactions with established experimental and experimental transferred evidence were filtered from the STRING database. Only PPIs with high confidence scores (totally 134 486 PPIs) were kept to ensure the network reliability and conciseness. With respect to regulatory interactions, 145 421 regulatory relationships were acquired from the RegNetwork database. Subsequently, the information obtained from all three types of interactions were incorporated into a comprehensive reference generic IN resulting in a total of 25 970 nodes and 295 668 edges and thereafter it is tailored into a HepG2-specific IN with totally 17 214 nodes and 185 133 edges. Note that the PPIs represent most relationships within the HepG2-specific IN (Supplementary Table S1).

**Figure 2.**
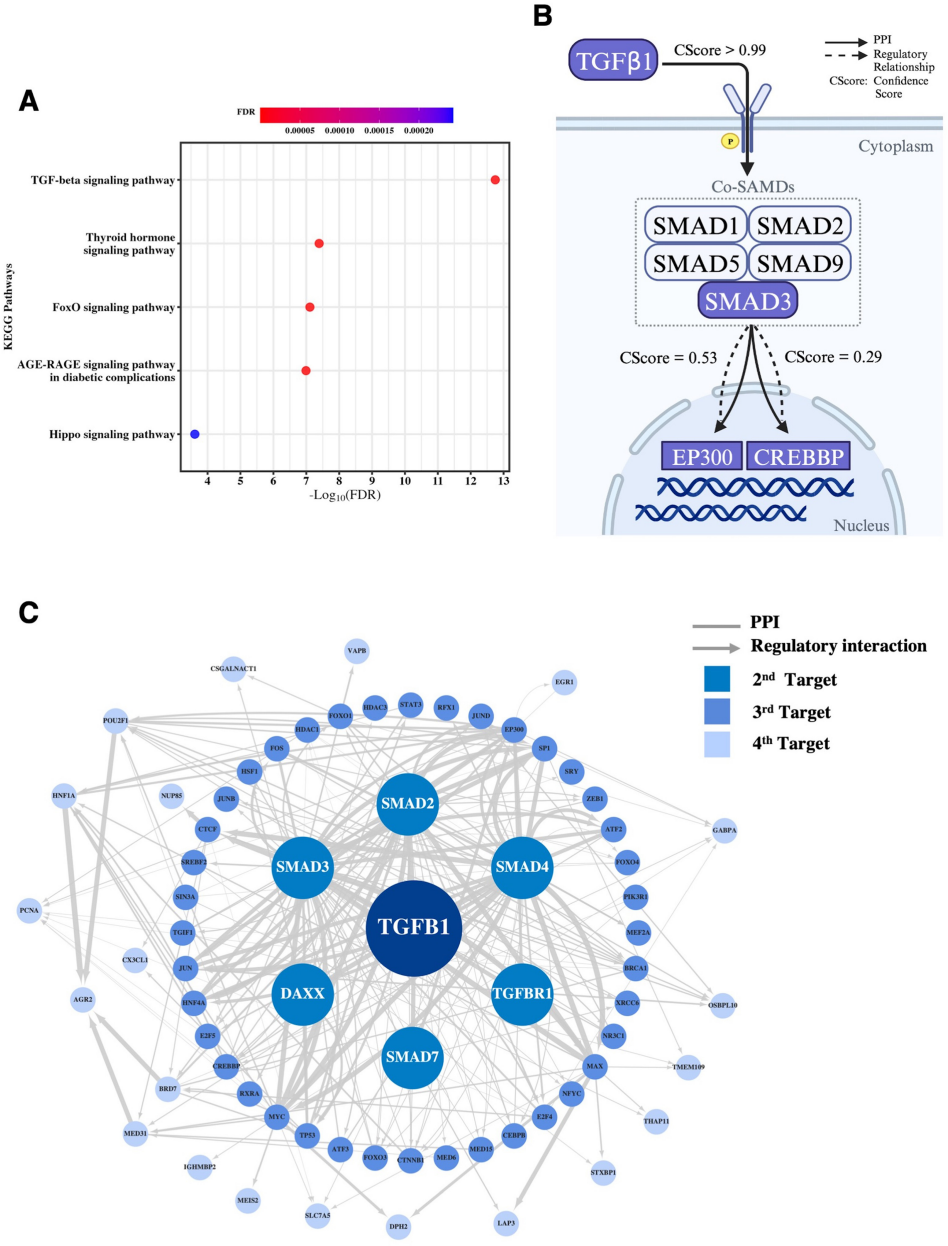
Validation of Open MoA in analyzing the key targets and reconstructing the TGF-β signaling pathway. (A) Top five signaling pathways in KEGG enrichment analysis of all predicted key targets in the TGF-β1 centric subnetwork (FDR<0.05) are presented. (B) Schematic diagram for the reconstruction of the TGF-β1 signaling pathway is shown. Targets involved in the pathways from TGF-β1 to gene EP300 or CREBBP are highlighted. (C) The TGF-β1 centric subnetwork. The second targets are those directly connected to TGF-β1. The third targets are the ones connecting the second targets, and others are shown. One interaction has no connection with the whole centric subnetwork; thus, it was deleted during visualization.

To demonstrate the capacity of Open MoA for identifying key intermediate TFs/genes and validate their usefulness, we first applied Open MoA to a TGF-β1 dataset to test if it can reconstruct the well-known TGF-β signaling pathway. The signature transcriptomic data of TGF-β1 including 12 205 genes with computed FDR values (Supplementary Table S1) that obtained from Connectivity Map (CMap) level 5th (Subramanian *et al.* 2017) was used for the subnetwork construction. More in details, gene perturbation values (*Z*-score) of HepG2 cell line after the treatment of TGF-β1 shRNA were converted into two-tailed *P*-values via *pnorm* function in R, followed by *p.adjust* function to calculate FDR values. Out of the 12 205 signature genes, 7524 genes could be mapped to nodes in the HepG2 reference IN (Fig. 1C). A TGF-β1-specific weighted subnetwork starts from TGF-β1 to its screened signature genes, with totally 23 466 edges owning the final confidence scores smaller than 1.00. The KEGG enrichment results for sensitivity analysis are shown in Supplementary Table S2. We found that the TGF-β signaling pathway is the top one signaling pathway in all centric subnetworks in terms of the FDR value and even the gene count. The result also suggests that Open MoA could effectively distinguish strong or weak correlations by assigning and computed confidence scores, and figure out the practical nodes and edges, which are related to TGF-β1 signaling pathway. Even the centric subnetwork consisted of edges with top 0.5% of confidence scores has the ability to display the most crucial and virtual biological pathways. Hence, the centric subnetwork with high confident edges that extracted from the TGF-β1-specific weighted subnetwork would facilitate clearer analyses and assist in exploring vital interactions in subsequent studies. Furthermore, in order to eliminate most of the disturbed noises while ensure greater retention of valid information and allow relative comparability between centric subnetworks, we defined the top 1% of confidence scores as the cutoff for screening the TGF-β1 centric subnetwork. Thus, edges with top 1% of confidence scores were extracted from the TGF-β1-specific weighted subnetwork to construct its centric subnetwork, which composes a total of 70 different nodes and 235 edges including 84 PPIs and 151 regulatory interactions (Supplementary Table S2). Besides, all nodes involved in the TGF-β1 centric subnetwork were also extracted and superimposed with pathways obtained from the KEGG database. Sixteen signaling pathways are significantly (FDR<0.05) enriched with genes in the TGF-β1 centric subnetwork in the KEGG pathway enrichment analysis (Supplementary Table S2). Among all these signaling pathways, the TGF-β signaling pathway is clearly the top one (FDR<10^−12^), followed by thyroid hormone signaling pathway (FDR<10^−7^), the FOXO signaling pathway (FDR<10^−7^), the AGE-RAGE signaling pathway in diabetic complications (FDR<10^−6^), and the Hippo signaling pathway (FDR<10^−3^) (Fig. 2A). Furthermore, we also mapped all identified nodes within the TGF-β1 centric subnetwork onto the TGF-β receptor signaling pathway of *H.sapiens* provided from WikiPathways. Here, we observed that key targets represented essential parts of the whole pathway, starting from TGF-β1 and its receptors to the SMAD complex (Shi *et al.* 2022), and signal eventually transmits into the nucleus for gene transcription (Supplementary Fig. S3A).

Figure 2C and Supplementary Table S2 display the key interactions within the TGF-β1 centric subnetwork, as well as the relationships between the TGF-β1 and SMAD family members, which show particular prominence out of the 23 466 relationships, accounting for extremely high confidence scores >0.99. SMAD family members are common targets of TGF-β1 in the signaling pathway that act as key TFs on following genes in nucleus (Dewidar *et al.* 2019). Interestingly, SMAD3 displays the most diverse interactions connecting 52 different downstream targets, while SMAD2 and SMAD4 have 36 and 40 downstream associations, respectively. However, there is no distinct difference in confidence scores between SMAD family members and their related proteins. Additionally, some other target related to the TGF-β1 signaling pathway, such as DAXX (Kim *et al.* 2021) also appeared in the centric subnetwork, supporting again the ability of Open MoA to identify key targets.

To demonstrate the capacity of Open MoA to uncover and dissect the core mechanisms underlying the observed biological processes, we reconstructed the exact TGF-β signaling pathway by measuring the most probable pathway between TGF-β and an ensemble of well-established downstream targets in the weighted TGF-β1 subnetwork. Open MoA predicted SMAD3 as the key intermediate regulator from TGF-β1 to EP300 (Ghosh and Varga 2007, Zoni *et al.* 2015) and from TGF-β1 to CREBBP (Abdel Mouti and Pauklin 2021), both results highly consistent with the reference signaling pathway and experimental evidence in the literature (Fig. 2B and Supplementary Table S2).

We also restricted the transcriptomic data from CMap database to all Best Inferred Genes only, and performed the analysis for TGF-β1 with same study design. Similar results are obtained and shown in Supplementary Table S4. Additionally, we have also performed similar analyses by Open MoA using transcriptomic signatures of WNT1 inhibition and metformin treatment. The results are also very promising which are shown in Supplementary Material S2.

### 3.2 Exploration of the MoA for target-oriented repurposed drugs

To show the predictive capability of the Open MoA, we ran our pipeline for revealing the MoA of a recently purposed drug based on network-based drug repositioning. We have previously established a computational pipeline for drug repurposing and identified JNK-IN-5A could modulate the expression of PKLR for effective treatment of non-alcoholic fatty liver disease (Zhang *et al.* 2022). JNK-IN-5A is known to bind c-Jun N-terminal kinases 3 (also known as MAPK10), however, its modulation on PKLR expression has not been reported yet. Hence, the investigation of how JNK-IN-5A modulates the mRNA expression of PKLR represents an interesting case for the application and evaluation of Open MoA.

Accordingly, we constructed a JNK-IN-5A-specific IN. Eleven thousand nine hundred thirty-six transcriptomic signature genes of JNK-IN-5A (10 μM, treated for 24 h) with associated *Z*-scores retrieved from CMap 5th level (Subramanian *et al.* 2017) were transferred into two-tailed *P*-values and finally FDR values by *pnorm* and *p.adjust* function separately as previously described (Supplementary Table S1). Only 7479 genes were matched with the HepG2-specific IN and followed by the implementation of MAPK10 and its DEGs as the starting point and endpoints, respectively. As a result, 7523 nodes and 23 697 edges were selected as activated in the JNK-IN-5A-specific IN. In addition, based on the feasibility of previous sensitivity analyses, edges with top 1% of confidence scores were selected for the construction of the JNK-IN-5A centric subnetwork, which comprised a total of 167 nodes and 237 edges (Supplementary Table S3).

**Figure 3.**
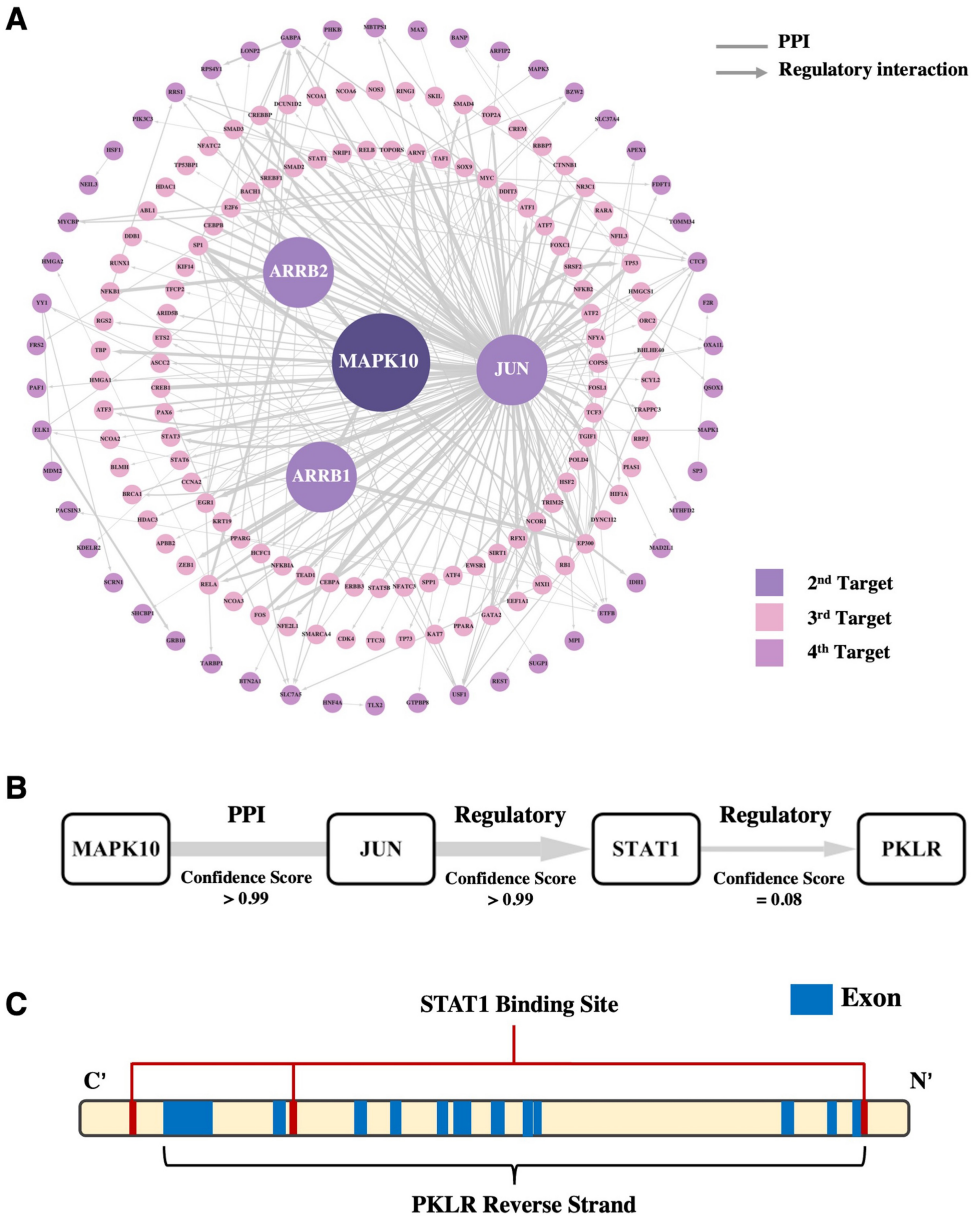
Prediction of MoA of JNK-IN-5A using Open MoA. (A) The JNK-IN-5A centric subnetwork is presented. The second targets are those directly connected to MAPK10. The third targets are the ones connecting the second targets, and others are shown. (B) Reconstruction of the WNT signaling pathway by Open MoA from MAPK10 to PKLR. (C) A schematic representation of the binding sites for the TF STAT1 on the PKLR gene based on GTRD.

JUN is a classical JNK substrate that can be phosphorylated by MAPK10 at its transactivation domain, especially at the serine residues S63 and S73 (Liu *et al.* 2006, Kunde *et al.* 2013), and participate in a large ensemble of cellular processes including cell proliferation, differentiation, migration, and apoptosis (Kunde *et al.* 2013, Zhu *et al.* 2014). Notably, as shown in Fig. 3A, though JUN, ARRB1, and ARRB2 are the direct downstream targets of MAPK10, the majority of nodes are only linked to JUN. It further highlights the predictive power of our pipeline. At the same time, MAPK10 may also have the potential to affect the expression level of genes encoding MYC, SP1, and TP53 due to their high edge confidence scores with JUN (Noguchi *et al.* 1999, Gao *et al.* 2016, Lee *et al.* 2020).

Subsequently, to predict the potential MoA of JNK-IN-5A in modulating the gene expression of PKLR, we set MAPK10 as the starting point and PKLR as the endpoint. Our analyses indicated that MAPK10 initially targets JUN and thereafter interacts with STAT1, resulting in an alternative PKLR expression modulation (Fig. 3B and Supplementary Table S3). Using the Gene Transcription Regulation Database (GTRD) (Yevshin *et al.* 2019), we also found three potential binding sites of STAT1 located close to PKLR in the genome, whose lengths are 76 bases, 76 bases, and 56 bases, respectively (Fig. 3C). Notably, only two of these binding sites are localized on the PKLR reverse strand. Therefore, we observed that the MoA of JNK-IN-5A in modulating the PKLR expression is promising.

### 3.3 STAT1 is the upstream TF of PKLR based on *in vitro* experiments

**Figure 4.**
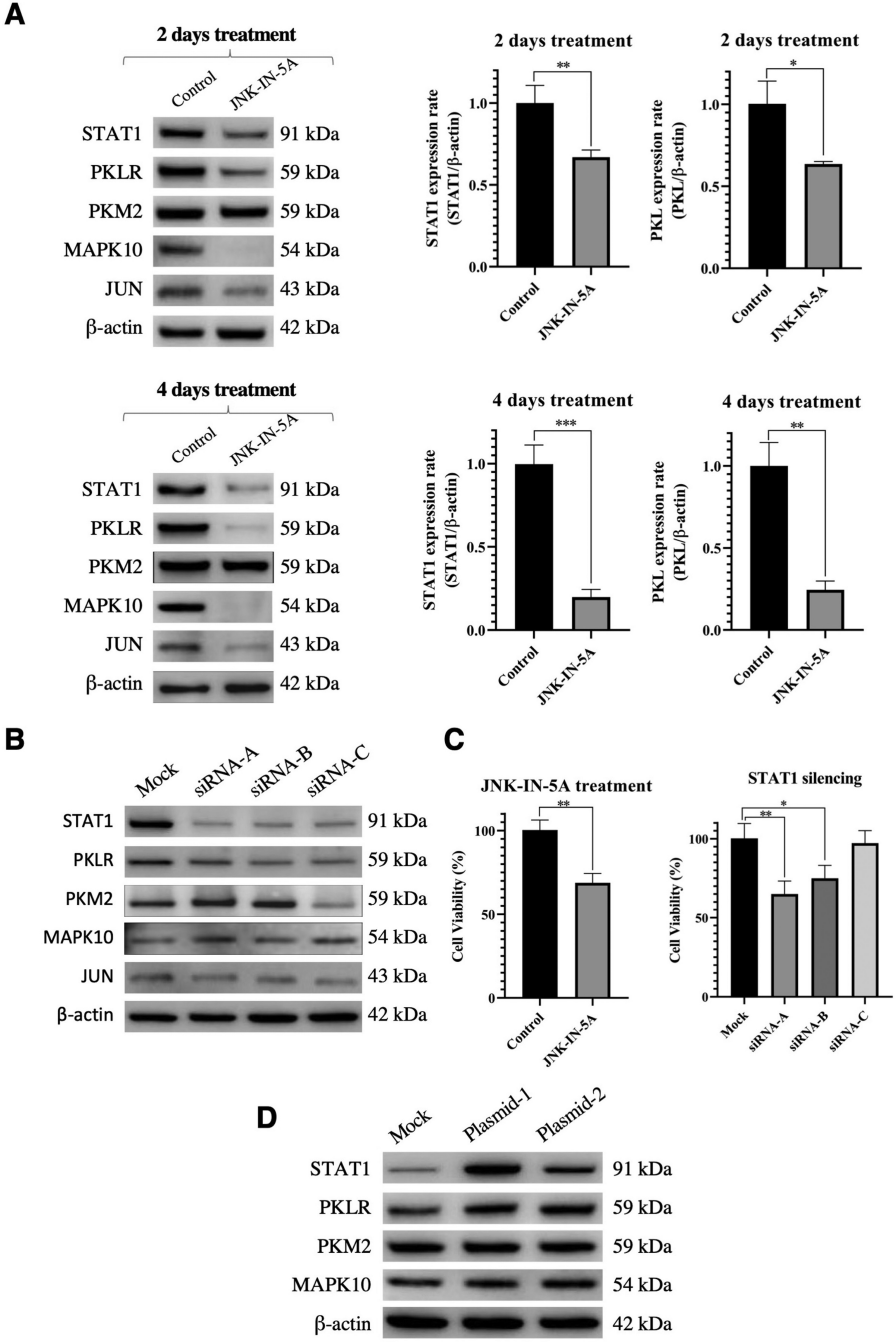
The effect of JNK-IN-5A on the target protein MAPK10 and downstream proteins (STAT-1, JUN, and PKLR) as a MoA prediction. (A) The expression levels of target and downstream proteins after treatment of 10 µM JNK-IN-5A in HepG2 cells for 2 and 4 days. β-actin was used as a loading control. Relative expression of STAT1 and PKL are presented as means ± SD from three independent experiments. (B) The effect of siRNA sequences of STAT1 (siRNA-A, siRNA-B, and siRNA-C) on the protein expressions. (C) The evaluation of the HepG2 cells viability after 2 days of the 10 µM JNK-IN-5A treatment and STAT-1 silencing. Cell viabilities are presented as means ± SD from triplicate measurement. (D) The expression level of the target and related proteins after 2 days treatment of STAT1 overexpression plasmids. Data information: In (A and C), Student’s *t-*test *P*-values indicate statistical significance, *P≤.05, **P≤.01, ***P≤.001.

To validate the effects of JNK-IN-5A on the target protein MAPK10 and downstream proteins (STAT1, JUN, and PKLR) predicted by Open MoA, we treated HepG2 cells with JNK-IN-5A and the expression levels of MAPK10 and measured the level of the proteins using western blot analyses (Fig. 4).

We first confirmed the efficacy of JNK-IN-5A by demonstrating that the expression levels of MAPK10 were significantly reduced after 2 days of JNK-IN-5A treatment (Fig. 4A). We also showed significant decreases in the STAT1 (Student’s *t*-test, *P*<.01) and PKLR (Student’s *t*-test, *P*<.05) expression levels in JNK-IN-5A-treated HepG2 cells after 2 days of the drug treatment (Fig. 4A). To examine the effect over a longer term, the HepG2 cells were treated with JNK-IN-5A for an additional 2 days. At the end of 4 days of treatment, our analyses demonstrated that the expression levels of STAT1 (Student’s *t*-test, *P* < .001) and PKLR (Student’s *t*-test, *P*<.01) decreased even more dramatically (Fig. 4A). These results suggested that STAT1 and PKLR are downstream targets of MAPK10. Notably, while the administration of JNK-IN-5A caused a significant decrease in PKLR expression via STAT1, we determined that PKM2, the isozyme of PKLR, was not affected by the administration of JNK-IN-5A.

Accordingly, we also treated HepG2 cells with three different siRNA sequences targeting STAT1 to examine the effects of STAT1 silencing on PKLR expression and other related proteins predicted by Open MoA. While there was a significant decrease in the PKLR protein levels in parallel with the decrease in STAT1 protein levels, siRNA treatment did not significantly affect the expression levels of MAPK10, PKM2, and JUN, as predicted (Fig. 4B). In addition, we tested the effects of siRNA and JNK-IN-5A treatments on cell viability by performing MTT tests. We observed that siRNA-A and siRNA-B treatments were approximately as effective as JNK-IN-5A on cell viability (Fig. 4C). Cell viability percentages of JNK-IN-5A, siRNA-A, siRNA-B, and siRNA-C groups were 68.8 ± 5.6, 65. ± 8.1, 74.9 ± 8.1, and 97.2 ± 7.9, respectively. Like the administration of JNK-IN-5A, silencing of STAT1 expression resulted in significantly decreased PKLR expression. In addition, MAPK10, JUN, or PKM2 showed no transcriptomic change with these siRNA administrations as predicted. Our analysis indicated that STAT1 is upstream of PKLR. It should also be noted that siRNA-C treatment was not as effective as siRNA-A and B on PKLR inhibition or cell viability, which is probably due to its low-binding efficiency.

Finally, we treated HepG2 cells with two different overexpression plasmids of STAT1 to test the effects of STAT1 overexpression on PKLR expression, and other related proteins identified through our MoA predictions. While we observed an increase in PKLR levels in parallel with increases in STAT1 levels, plasmids treatment for overexpression of STAT1 did not affect MAPK10 and PKM2 expression levels (Fig. 4D). In summary, we predicted the MoA of JNK-IN-5A and validated our findings by performing *in vitro* experiments.

## 4 Discussion

In this study, we proposed a computational pipeline based on the weighted IN that allows the users to predict a possible underlying MoA for the repurposed drugs and to identify potential drug targets. We successfully reconstructed the TGF-β signaling pathway (Fig. 2), the WNT signaling pathway (Supplementary Material S2), and the metformin subnetwork (Supplementary Material S2) through Open MoA. The results all demonstrated its capability to both identify key targets and predict prospective pathways of biological processes. Furthermore, we explored the potential MoA of repurposed drugs (JNK-IN-5A) using Open MoA and predicted that it modulates PKLR expression via MAPK10-JUN-STAT1-PKLR pathway. Importantly, our experimental results confirmed that targeting MAPK10, JNK-IN-5A resulted in the decreased protein expression levels of JUN, STAT1, and PKLR. In addition, we showed that the inhibition and overexpression of STAT1 respectively decreased and increased the expression of PKLR without major expression changes of MAPK10 or JUN, which indicates that STAT1 is the upstream TF of PKLR and downstream of JUN and MAPK10. Notably, the inhibition of PKLR induced by siRNA of STAT1 is not as much as the decrease caused by JNK-IN-5A. This suggests that JNK-IN-5A could have affected other regulators of PKLR that have combinatory inhibition effect on it. It should be noted that the PScore in the method only measures the probability but not the contributions. However, it is also possible that some pathways with suboptimal PScores have more significant influence on the targets. Therefore, other pathways, which have less confidence scores than the optimal one could be also interesting as they might reveal the other important TFs potentially regulated PKLR expression together with STAT1.

Our computational pipeline can predict the drug MoAs at both molecular and genetic levels. The “Drug-Protein-Gene” motifs it portrays could also infer the key regulatory interactions, such as the one presented in our study. Although a growing number of drug repurposing approaches provide insights into the potential MoAs (Zhang *et al.* 2020, Groza *et al.* 2021, MotieGhader *et al.* 2022), most of them were using clustering methods to show the similarities between drugs and diseases without revealing the step-by-step connection of the MoAs. In contrast, Open MoA would be good at facilitating the development of small molecular drug repurposing, since it reveals the MoAs of repurposed drugs stretching from general cellular and molecular levels to highly specific genetic levels following the basis of “Drug-Protein-TF-Gene” biological theory. Therefore, Open MoA represents a potent tool for the discovery of the MoAs of repurposed drugs.

Although we demonstrated that Open MoA could provide practical suggestions for the step-by-step mechanism of drugs or gene modulation, the key assumptions the method built on are not always true which could lead to some limitation of its application. First, the reference IN is not exhaustive, so the current version of the method may not be able to identify unknown MoAs or the ones which are not included in the scope of the IN, such as metabolic interactions or miRNA regulation. Therefore, one of the future directions of Open MoA could be enlarging the reference IN by increasing the quantity and diversity of biological data, such as metabolomics and miRNA (Ferragut Cardoso *et al.* 2020, Wu *et al.* 2020). In addition, there is a potential literature bias in the IN, as poorly studied genes/proteins are less likely to appear in the results. Thus, continuous updating and inclusion of data from different resources for the reconstruction of IN is crucial for the robustness of this method.

Moreover, our assumption about the shortest path within the IN can be contentious given that biological processes might not always follow the most direct routes. Yet, when exploring previously undiscovered MoA within vast biological networks, this approach proves relevant. Shortest path analysis has already been a common method for biological research (Kuperstein *et al.* 2015), including PPI network analysis and gene co-expression network analysis. In PPI networks, the shortest path analysis is used for indicating proteins with similar functions or functional protein complexes in biological pathways (Li *et al.* 2012, Badkas *et al.* 2022). For the gene co-expression networks, the shortest paths could separate gene modules since genes may closely connect to each other when they have identical functions (Cerami *et al.* 2010, Gu *et al.* 2010). Analogue principle has also been applied in the pharmaceutical networks to explore the relationship between drug and disease modules (Yang *et al.* 2018, Wei *et al.* 2021) and in disease networks to identify common candidates across separate disease modules (Garcia-Vaquero *et al.* 2018). In our research, the “guilt by association” heuristic anchors the shortest path analysis. It does not cluster based on similarity but discerns significance and specificity within the complex biological networks, making it pivotal in projecting potential drug MoAs.

In summary, Open MoA that emphasizes the regulatory relationships is a powerful network-based method for unveiling the potential fundamental MoA of the repurposed drugs and inferring the key drug candidates. By inputting original FDR values, Open MoA could give out the significant confidence score for each interaction and calculate the most possible pathway of drug action. When further discovering the underlying MoA of PKLR modulation by JNK-IN-5A from our previous study, we found that STAT1 is the essential upstream TF, which regulates PKLR expression. Moreover, the applications of Open MoA are not limited in the drug discovery, since it could also facilitate other mechanistic studies, which searches potential pathways of specific gene modulations as exemplified in the WNT1 and TGF-β1 cases in this study. Collectively, Open MoA is a promising tool for shedding light on drug repurposing by providing holistic understanding of the MoA and identifying novel drug targets.

## Supplementary Material

btad666_Supplementary_Data

## Data Availability

The source code of Open MoA is available at https://github.com/XinmengLiao/Open_MoA.
