# Supplementary material for: Open MoA: revealing the mechanism of action (MoA) based on network topology and hierarchy
Source: Bioinformatics. 2023 Oct 31;39(11):btad666. doi: 10.1093/bioinformatics/btad666 (PMC10637856; doi:10.1093/bioinformatics/btad666)
Supplement: btad666_Supplementary_Data [file btad666_supplementary_data.zip › Open_MoA_Supplementary_revised_10.19.docx]

**Supplementary Materials**

**S1 Supplementary Methods**

**S1.1 Differentially expressed genes (DEGs) collection and false discovery rate (FDR) calculation**

Signature transcriptomic data were downloaded from 5th level Connectivity Map (CMap) (**Subramanian *et al,* 2017**). Gene perturbation values (z-score) of HepG2 cell line after the treatment of TGF-β1 shRNA, WNT1 shRNA, and JNK-IN-5A (10 μM, treated for 24hr) were converted into two-tailed p-values via *pnorm* function in R, followed by *p.adjust* function to calculate FDR values respectively. For metformin, raw RNA sequencing data of HepG2 cell line and those treated with metformin are downloaded from GSE179347 dataset in Gene Expression Omnibus (GEO). Samples GSM5415748, GSM5415749, and GSM5415750 were assigned as the control group, while samples GSM5415790, GSM5415791, and GSM5415792 were used as the treatment group. The low expression genes with counts less than 1.00 were filtered out firstly. FDR value of each metformin DEG were computed by the statistics differences between control group and treatment group based on the gene counts via DESeq2 v1.34.0 in R.

**S1.2 Construction of the weighted subnetworks for** **TGF-β1, WNT1, metformin, and JNK-IN-5A**

The TGF-β1 weighted subnetwork was constructed in Open MoA by setting TGF-β1 as the starting point and its screened DEGs as the endpoints. As illustrated in **Fig. 1C**, the TGF-β1 signature genes (DEGs) were mapped to nodes in the HepG2 reference IN. All shortest paths with minimal edge numbers between TGF-β1 and their signature genes were identified by method **2.3**. Next, each edge included in the shortest path was assigned with the confidence score, which is calculated by **Equation (2)**, **Equation (3)**, and **Equation (4)**. Hence, a TGF-β1-specific weighted subnetwork was generated with edges having different confidence scores that present their opportunities to be activated. The WNT1, metformin, and JNK-IN-5A weighted subnetworks are built in the same way by setting WNT1, metformin, and MAPK10 as the starting point and their screened DEGs as the endpoints separately.

## S1.3 Reconstruction of TGF-β and WNT signaling pathways

The core pathways with the highest confidence score from TGF-β1 and WNT1 to their targeted genes were reconstructed from their respective specific weighted subnetworks by the shortest path function in igraph v1.3.5 in R. The schematic diagrams of TGF-β1 and WNT1 reconstructed signaling pathways were created by BioRender.com (2023).

## S1.4 Prediction of JNK-IN-5A MoA

To predict one of the underlying MoAs of drug repurposing for PKLR modulation by JNK-IN-5A, MAPK10 was applied as the starting point while DEGs under JNK-IN-5A intervention obtained from the CMap were set as the endpoints. All original FDR values of these DEGs were then calculated through Open MoA to generate a JNK-IN-5A-specific weighted subnetwork. Finally, the key pathway with the highest confidence score between MAPK10 and PKLR representing the most possible MoA of JNK-IN-5A was predicted by the shortest path function in igraph v1.3.5 and visualized via Cytoscape. Additionally, to further verify the regulatory interaction between STAT1 and PKLR, transcription factor binding sites of STAT1 in gene PKLR were found in Gene Transcription Regulation Database (GTRD) v21.12 (**Yevshin *et al,* 2018**). The advanced search was used by choosing Homo Sapiens to be the organism and meta cluster to be the dataset, setting PKLR to be gene symbol and STAT1 (P42224) to be the transcription factor, keeping the default max gene distance as 5000.

**S1.5 Sensitivity Analysis and the construction of centric subnetworks**

Sensitivity analysis is used for selecting the proper cutoff for the centric subnetworks. Within the context-specific weighted subnetwork, edges with top 0.5%, 1%, and 2% of confidence scores were extracted to generate the context-specific centric subnetworks separately. KEGG enrichment analysis was used to further explore and compare the functions of key targets involved in the centric subnetworks. OMIM analysis was furthermore conducted on metformin centric subnetworks. The context centric subnetworks were then built under the selected threshold and visualized by Cytoscape v3.9.1 (**Kohl *et al,* 2011**).

## S1.6 KEGG Functional enrichment analysis

To further explore the functions of key targets and interactions presented in the TGF-β1 weighted subnetworks, DAVID (the Database for Annotation, Visualization and Integrated Discovery website) (**Dennis *et al,* 2003**) was utilized to conduct the Kyoto Encyclopedia of Genes and Genomes (KEGG) (**Kanehisa *et al,* 2023**) enrichment analysis on the core targets. Therefore, all nodes from the context centric subnetworks were extracted separately and imported into the DAVID database to analyse with the functional annotation tool respectively. The following operations were conducted, choosing the official gene symbol to be the identifier as well as Homo sapiens to be the species, and setting the gene list to be the list type. The results of KEGG pathways were generated and downloaded from the DAVID website. FDR value < 0.05 was used to indicate statistical significance. Top 5 signaling pathways of TGF-β1 relevant nodes were visualized in bubble plots by ggplot2 v3.3.5 in R.

## S1.7 Online Mendelian Inheritance in Man (OMIM) analysis

To cluster all nodes in metformin centric subnetwork, we mapped them with disease-gene association data from OMIM knowledgebase (**Amberger *et al,* 2017**) by Enrichr v3.1 in R. The following steps were conducted, setting Enrichr website to human genes, choosing OMIM disease database as the background database, and enriching the key nodes into the background database. FDR  < 0.05 was used to indicate statistical significance. Top 5 disease terms were visualized in bubble plots by ggplot2 v3.3.5 in R. All the plots were rearranged by patchwork v1.1.3 in R.

**S1.8 Reproducing key targets in the TGF-β1 and WNT1 signaling pathway**

For the sake of verifying the accuracy of the subnetworks constructed via Open MoA, all nodes involved in the TGF-β1 and WNT1 centric subnetworks were mapped and portrayed with the TGF-beta receptor signaling (Homo sapiens) (WikiPathways WP560) and WNT signaling (Homo sapiens) (WikiPathways WP428) separately in WikiPathways (**Slenter *et al,* 2018**) using rWikipathways package v1.14.0 in R.

**S1.9 Cell culture and transfection of siRNA and cloned vector**

The HepG2 cells were maintained in RPMI-1640 medium (R2405, Sigma Aldrich, USA) supplemented with 10% fetal bovine serum (FBS) and 1% penicillin/streptomycin (P/S) solution in a humidified incubator with 5% CO2 at 37°C. For the drug treatment, cells were seeded in 6-well plate at 2.5x105 cells. After 24 hours of cell seeding, 10 µM JNK-IN-5A (HY-15881, MedChemExpress, USA) dissolved in Dimethyl sulfoxide (DMSO, 41639, Sigma-Aldrich) was treated to the cells for two and four days with 0.1% final DMSO concentration. For siRNA treatments of STAT1, 2.5x105 cells were seeded in a 6-well plate to each well. After 24 hours of cell seeding, 25 pmol STAT1 human siRNA Oligo duplexes (SR321905, Origene) were transfected by Lipofectamine® RNAiMAX (13778-075, Thermo Fisher Scientific, USA) for 2 days. For overexpression of STAT1, we constructed two expression vectors of STAT1 (STAT1-201 and STAT1-226 coding sequences) via GenScript Company (Nanjing, China). For the treatment of overexpression vectors, 2.5x105 cells were seeded to per well in 6-well plate. After 24 hours of cell seeding, 5 µg overexpression vectors of STAT1 were transfected by Lipofectamine™ 3000 Transfection Reagent (L3000001, Thermo Fisher Scientific, USA) for two days. It was applied with the same transfection agents in mock groups for negative control of siRNA and overexpression applications.

## S1.10 Western Blot

The cells were washed with phosphate-buffered saline (PBS) and lysed with CelLytic M (C2978, Sigma-Aldrich) lysis buffer containing protease inhibitors (11836170001, Roche, Switzerland). The cell lysates were centrifuged at 12,000 rpm for 10 min and supernatants were collected. Protein Assay Dye Reagent (5000006, Bio-Rad) was used for the determination of protein concentration. The absorbance of the proteins was measured spectrophotometrically at 595 nm by a microplate reader (Hidex Sense Beta Plus). SDS PAGE was performed using Mini-PROTEAN® TGXTM Precast Gels (4561086, Bio-Rad, USA) and then the separated proteins were transferred to a Trans-Blot Turbo Mini 0.2 um PVDF Transfer Packs membrane (1704158, Bio-Rad, USA) by using Trans-Blot® TurboTM Transfer System (Bio-Rad, USA). The membrane was blocked with 5% skim milk for 30 min at 4°C with gentle rocking. After blocking, the membrane was treated with primary antibodies: Anti-Stat1 (HPA000982, Atlas Antibodies, Sigma-Aldrich, USA), Anti-JNK3 (MA5-35246, Invitrogen, USA) and Anti-c-Jun (HPA059474, Atlas Antibodies, Sigma-Aldrich, USA) and Anti-Beta actin (ab8227, Abcam, UK) as an internal control, overnight at 4°C on a rocking platform. After the treatment of primary antibodies, the membrane was washed three times with TBS-T buffer (A09-7500-100, Medicago, Sweden). Then, Goat anti-Rabbit IgG-HRP was treated as the secondary antibody for 30 min at 4°C with gentle rocking. The protein bands on the membrane were revealed using enhanced chemiluminescence substrate (WBLUF0500, Merck, USA) and detected with ImageQuant™ LAS 500 (GE Healthcare, USA).

**S2 Supplementary Results**

## S2.1 Reconstruction of the WNT Signaling Pathway by Open MoA

To further evaluate the power of Open MoA, we used the same approach for the TGF-β1-specific weighted subnetwork described above and reconstructed a weighted subnetwork for WNT1 inhibition in the HepG2 cell line. The WNT signaling pathway is highly conserved and commonly activated in various kinds of liver cancers (**He *et al,* 2020**). In the context of the WNT signaling pathway, 7.523 out of 12,213 transcriptomics signature genes with transferred FDR values following WNT1 shRNA inhibition in HepG2 cells (**Supplementary Table 1**) were mapped onto the HepG2-specific IN. Consequently, WNT1 and its DEGs with FDRs were imported into Open MoA as starting point and endpoints respectively for confidence score calculation. A total of 26,360 interactions were computed and assigned with the potential activation (indicated by nonzero confidence scores) in the WNT1-specific weighted subnetwork.

Based on the KEGG enrichment results of sensitivity analysis in **Supplementary Table 2**, we found that no matter under which threshold, the centric targets most significantly concentrated in WNT signaling pathway (FDR < 0.05). Therefore, for clearly visualizing and comparing the centric subnetwork, we still choose edges with top 1% of confidence scores to build the WNT1-specific weighted subnetwork. It is comprised of 126 nodes and 264 edges (**Supplementary Table 2**). KEGG enrichment analysis was then conducted on all involved nodes to investigate their functional relevance. Among the 29 most prominent signaling pathways (FDR < 0.05) in our KEGG results (**Supplementary Table 2**), the most significant pathway was the WNT signaling pathway (FDR < 10^-11^). The other top five enriched KEGG pathways are TGF-β signaling pathway (FDR < 10^-10^), the AGE-RAGE signaling pathway (FDR < 10^-7^), hippo signaling pathway (FDR < 10^-6^), and the thyroid hormone signaling pathway (FDR < 10^-6^) (**Supplementary Fig. S1A**). Furthermore, we mapped all nodes from the WNT1 centric subnetwork onto the reference WNT signaling pathway of Homo Sapiens from the WikiPathways database. We found that the key targets including GSK3B, LRP5, LRP6 and CTNNB1 are mostly participating in the main part of WNT signaling pathway (**Supplementary Fig. S3B**).

When looking into the WNT1 centric subnetwork, we observed that GSK3B, LRP5, and LRP6 are all key direct targets of WNT1 and all downstream processes pass through these three targets (**Supplementary Fig. S1C**). Notably, out of 8 targets that could be potentially activated by WNT1 in the WNT1-specific weighted subnetwork, these three targets displayed the highest confidence scores in the entire centric subnetwork, indicating their importance for function of WNT. Furthermore, several other well-known downstream targets of WNT such as EGR1 (**Lei *et al,* 2019**) and E2F1(**Calvisi *et al,* 2005**) were also identified by our analyses in the centric subnetwork.

Genes CCND1 and CCND3 are reported as the targeted genes of the the canonical WNT signaling pathway (**DiMeo *et al,* 2009; Röhrs *et al,* 2009**). MYC is also a key downstream effector of beta-catenin in the WNT signaling pathway (**Xu *et al,* 2022**), whose aberrant activation can lead to the progression of undifferentiated hepatocellular carcinoma (HCC) (**Bisso *et al,* 2020**). Therefore, to validate the function of Open MoA and predict the precise biological process, we tried to reconstruct the signaling pathway from WNT1 to CCND1, CCND3, and MYC. As displayed in **Supplementary Fig. S1B** and **Supplementary Table 2**, the predicted pathway starts from WNT1, then continues through the LRP5, GSK3B-CTNNB1 complex, and SMAD3, finally reaches CCND1 and CCND3. However, for MYC as the endpoint, TP53 is showed up to be the last TF that regulates MYC expression. The low-density lipoprotein receptor-related proteins 5/6 (LRP5/6) are typical receptors on plasma membrane that play crucial roles in the initiation of WNT signaling transduction (**Hua *et al,* 2018**). The CTNNB1 encodes the protein beta-catenin (**He *et al,* 2020**) and GSK3B encodes a part of a protein complex, which recruits and phosphorylates beta-catenin, leading to the inhibition of HCC pathogenesis (**Chen *et al,* 2017**). Collectively, LRP5/6, GSK3B and CTNNB1 are all three very common intermediate targets and key components of the WNT signaling pathway. Thus, the upstream pathway is similar to the canonical WNT signaling pathway (**Katoh *et al,* 2007**). The reconstructed pathways towards CTNND1 and CTNND3 are both agree with the reference pathways and experimental evidence in the literatures (**Bennett *et al,* 2010; Xia *et al,* 2019**). Although TP53 does not directly involved in the WNT signaling process, it actively interacts with the beta-catenin destruction complex and other elements of WNT signaling pathway in cancers (**Xiao *et al,* 2022**). Meanwhile, TP53 could represses c-myc function by binding its promoter (**Ho *et al,* 2005**), thus, the MYC-induced apoptosis could be enforced via the p14ARF/p53 axis (**McMahon 2014**). Hence, the confidence score of edge form TP53 to MYC is equal to 0 may indicate this regulatory interaction has little correlation with WNT-related biological reaction. The appearance of this relationship might be caused by the high connection between GSK3B and TP53. However, based on the roles that WNT and TP53 pathways playing in cancer progression, TP53 may have the potential in modulating MYC expression by interacting with WNT signaling pathway. Further studies are needed for investigation.

## S2.2 Construction of the metformin subnetwork by Open MoA

To test the performance of Open MoA in predicting the MoA of drugs, transcriptomics data before and after the treatment of metformin were downloaded from GEO and different expression analyses were performed to obtain the DEGs based on FDRs. A total of 8,320 DEGs were obtained (**Supplementary Table 1**) and 7,283 of these were mapped onto the HepG2-specific IN. During the confidence score calculation, 7,343 nodes and 19,327 edges were computed and assigned with different computed FDR values which together compose the metformin-specific weighted subnetwork. Likewise, from the sensitivity analysis (**Supplementary Table 3**), we extracted the edges with top 1% of confidence scores in the metformin-specific weighted subnetwork to generate a metformin centric subnetwork, which consists of 132 nodes and 194 edges (**Supplementary Table 3**). Moreover, all nodes in the centric subnetwork were compared with gene sets in the KEGG pathway database as well as the OMIM disease database to assess their main biological function(s) (**Supplementary Table 3**). The key targets are significantly enriched in 84 different KEGG pathways (FDR < 0.05) and are mostly concentrated in pathways associated with cell physiological activities, viral infections, inflammations, and cancers including leukemia, pancreatic cancer and prostate cancer (**Supplementary Table 3**). More specially, 17 key genes are involved in the longevity regulating pathway (FDR < 10^-17^) (**Supplementary Fig. S2A**). Metformin is a well-known drug that is widely used for treating type 2 diabetes mellitus, and this is in agreement with our results, where type 2 diabetes is enriched significantly (FDR < 0.05) as shown in **Supplementary Fig. S2B**. In addition, many of the biological processes in OMIM database, such as leukemia (**Biondani *et al,* 2018**), breast cancer (**Cejuela *et al,* 2022**), immunodeficiency (**Ouyang *et al,* 2020**), and obesity (**Yerevanian *et al,* 2019**) (**Supplementary Fig. S2B**) are associated with central genes in the metformin centric subnetwork. Taken together, we observed that Open MoA can predict the metformin centric subnetwork in a very good agreement with the literatures.

Although metformin has three targeted proteins which are encoded by PRKAB1, GPD1, and ETFDH in the metformin specific weighted subnetwork, our analyses indicated that only targets encoded by gene PRKAB1 is the downstream targets in its centric subnetwork (**Supplementary Fig. S2C, Supplementary Table 3**). The confidence score of the metformin and PRKAB1 interaction is infinitely close to 1.00, and importantly, this result reveals the MoA of metformin. PRKAB1 is one of the genes that encodes the AMP-activated protein kinase (AMPK) which is involved in one of the principal mechanisms underlying metformin activity (**Zhou *et al,* 2022**). It has been previously reported that activation of AMPK by metformin could boost mitochondrial respiration and restore the mitochondrial life cycle, which contributes to the reduction of oxygen consumption (**Wang *et al,* 2019**) and improvement of liver diseases (**Zheng *et al,* 2013**). AMPK subunits are also encoded by genes PRKAA1, PRKAA2, PRKAG1, PRKAB2, PRKAG2, and PRKAG3, while three of them show close connections with PRKAB1 (**Supplementary Fig. S2C**). Therefore, the only downstream target of PRKAB1 is ULK1. The confidence score of PRKAB1-ULK1 relationship is nearly 1.00, which is also corresponding with the studies (**Hsieh *et al,* 2017**). Furthermore, TP53 is identified as the most connected intermediate transcription factor in metformin centric subnetwork (**Supplementary Fig. S2C**), which suggests its key role in the MoA of metformin. Previous study also supported that metformin could enhanced TP53 transactivation activity and influence various downstream targets to suppress HCC cell growth and survival (**Yi *et al,* 2019**). Altogether, Open MoA analyses indicate that the metformin centric subnetwork represents the main AMPK-dependent mechanism of metformin action, which could modulate the downstream gene expression probably by the ‘AMPK-ULK1-HTT-TP53’ pathway.

**S3 Supplementary figures**


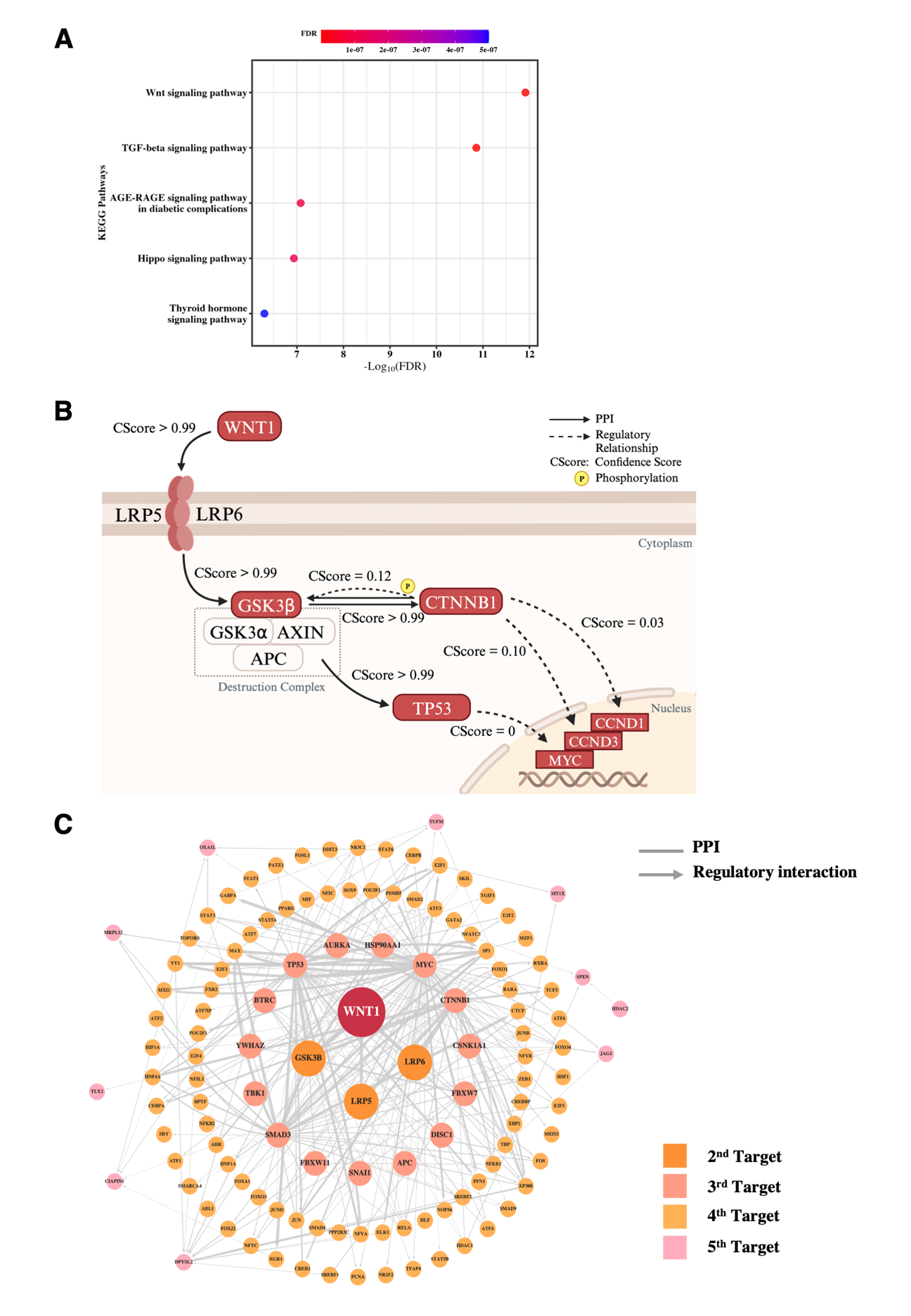


**Fig. S1** **Validation for Open MoA in analyzing the key targets and reconstructing the signaling pathway of WNT.**

(A) Top 5 signaling pathways in KEGG enrichment analysis of all key targets in WNT1 centric subnetwork (FDR < 0.05) are presented.

(B) Schematic diagram for the reconstruction of the WNT1 signaling pathway. Targets involved in the pathways from WNT1 to genes MYC, CCND1, and CCND3 are highlighted with color red.

(C) WNT1 centric subnetwork. The 2^nd^ targets are those directly connect to WNT1. The 3^rd^ targets are the ones connecting the 2^nd^ targets, and others are shown. Additionally, two interactions and four nodes are not connected to the WNT1 centric subnetwork, thus they were deleted during visualization.


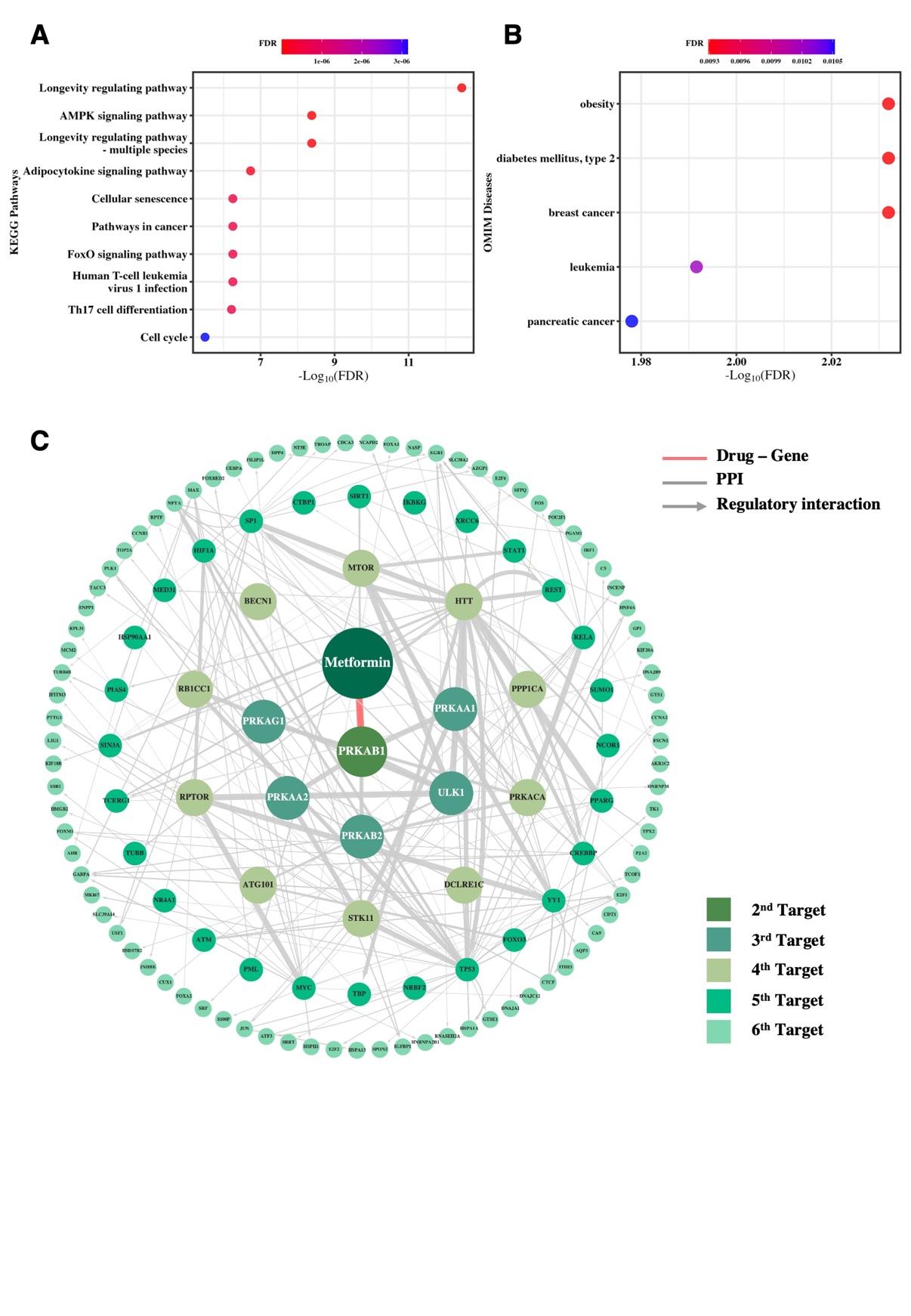


**Fig. S2 Validation for Open MoA in analyzing the key targets of metformin.**

(A) Top 10 pathways (FDR < 0.05) in KEGG enrichment analysis of all key targets in metformin centric subnetwork are presented.

(B) Top 5 diseases (FDR < 0.05) in OMIM Disease database enrichment analysis of all targets in metformin centric subnetwork are presented.

(C) The metformin centric subnetwork. The 2^nd^ targets are those directly connect to metformin. The 3^rd^ targets are the ones connecting the 2^nd^ targets, and others are shown. Two interactions and four nodes were removed since their independency from the metformin centric subnetwork.


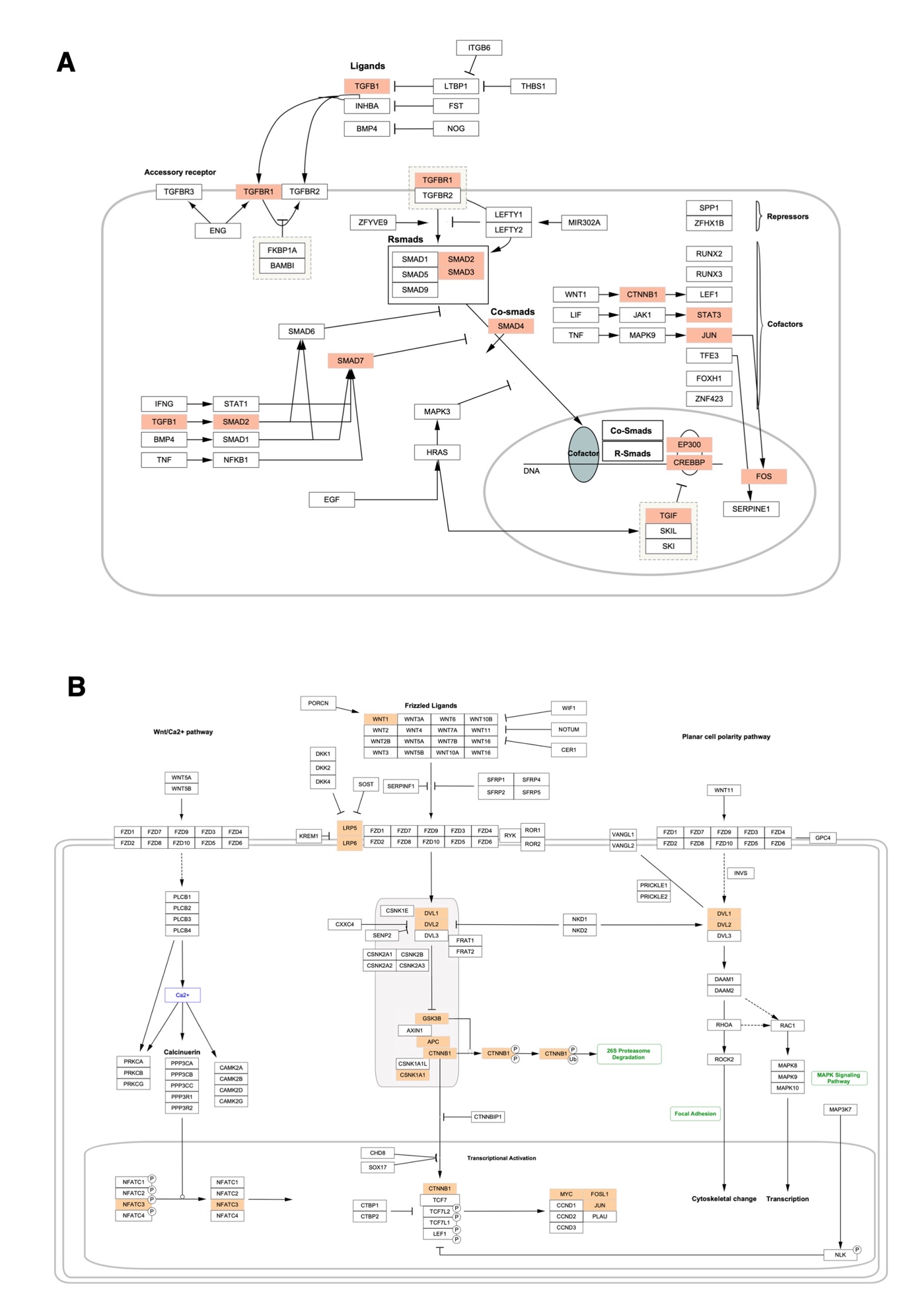


**Fig. S3 The locations of key targets in reference signaling pathways.**

After mapping of all key targets from the TGF-β1 and WNT1 centric subnetworks to their reference signaling pathways, orange targets in (A) show the locations of matched targets of TGF-β1 signaling pathway, while the orange targets in (B) show the matched targets of WNT1 signaling pathway.

**Table S1 The details of three interactions in the HepG2 specific network**

| Interaction type | Description | Number | |
| --- | --- | --- | --- |
|  |  | Node | Edge |
| Drug-Gene | The drug-gene interactions are the connections of drugs and their detailed target genes. All these interactions in the reference network are from the DrugBank database. | 8,574 | 16,571 |
| TF-Gene | The transcription factor-gene interactions are combination of TFs and their downstream regulated genes. All these interactions included in the reference network are from the RegNetwork database. | 9,995 | 72,238 |
| Protein-Protein | The protein-protein interactions are highly specific physical contacts between two or more proteins. PPIs in the reference network are from the STRING databases. | 6,285 | 96,324 |

**Reference**

Amberger JS, Hamosh A. Searching online mendelian inheritance in man (OMIM): a knowledgebase of human genes and genetic phenotypes. *Current protocols in bioinformatics* 2017;**58:** 1.2. 1-1.2. 12.

Bennett LB, Taylor KH, Arthur GL*, et al*. Epigenetic regulation of WNT signaling in chronic lymphocytic leukemia. *Epigenomics* 2010;**2:** 53-70.

Biondani G, Peyron JF. Metformin, an Anti-diabetic Drug to Target Leukemia. *Front Endocrinol (Lausanne)* 2018;**9:** 446.

Bisso A, Filipuzzi M, Gamarra Figueroa GP*, et al*. Cooperation Between MYC and β-Catenin in Liver Tumorigenesis Requires Yap/Taz. *Hepatology* 2020;**72:** 1430-1443.

Calvisi DF, Conner EA, Ladu S*, et al*. Activation of the canonical Wnt/beta-catenin pathway confers growth advantages in c-Myc/E2F1 transgenic mouse model of liver cancer. *J Hepatol* 2005;**42:** 842-849.

Cejuela M, Martin-Castillo B, Menendez JA*, et al*. Metformin and Breast Cancer: Where Are We Now? *Int J Mol Sci* 2022;**23:**

Chen J, Rajasekaran M, Hui KM. Atypical regulators of Wnt/β-catenin signaling as potential therapeutic targets in Hepatocellular Carcinoma. *Exp Biol Med (Maywood)* 2017;**242:** 1142-1149.

Dennis G, Sherman BT, Hosack DA*, et al*. DAVID: database for annotation, visualization, and integrated discovery. *Genome biology* 2003;**4:** 1-11.

DiMeo TA, Anderson K, Phadke P*, et al*. A novel lung metastasis signature links Wnt signaling with cancer cell self-renewal and epithelial-mesenchymal transition in basal-like breast cancer. *Cancer research* 2009;**69:** 5364-5373.

He S, Tang S. WNT/β-catenin signaling in the development of liver cancers. *Biomed Pharmacother* 2020;**132:** 110851.

Ho JS, Ma W, Mao DY*, et al*. p53-Dependent transcriptional repression of c-myc is required for G1 cell cycle arrest. *Mol Cell Biol* 2005;**25:** 7423-7431.

Hsieh FS, Chen YL, Hung MH*, et al*. Palbociclib induces activation of AMPK and inhibits hepatocellular carcinoma in a CDK4/6-independent manner. *Mol Oncol* 2017;**11:** 1035-1049.

Hua Y, Yang Y, Li Q*, et al*. Oligomerization of Frizzled and LRP5/6 protein initiates intracellular signaling for the canonical WNT/β-catenin pathway. *J Biol Chem* 2018;**293:** 19710-19724.

Kanehisa M, Furumichi M, Sato Y*, et al*. KEGG for taxonomy-based analysis of pathways and genomes. *Nucleic Acids Res* 2023;**51:** D587-d592.

Katoh M, Katoh M. WNT signaling pathway and stem cell signaling network. *Clin Cancer Res* 2007;**13:** 4042-4045.

Kohl M, Wiese S, Warscheid B. Cytoscape: software for visualization and analysis of biological networks. *Methods Mol Biol* 2011;**696:** 291-303.

Lei T, Zhu X, Zhu K*, et al*. EGR1-induced upregulation of lncRNA FOXD2-AS1 promotes the progression of hepatocellular carcinoma via epigenetically silencing DKK1 and activating Wnt/β-catenin signaling pathway. *Cancer Biol Ther* 2019;**20:** 1007-1016.

McMahon SB. MYC and the control of apoptosis. *Cold Spring Harb Perspect Med* 2014;**4:** a014407.

Ouyang J, Isnard S, Lin J*, et al*. Metformin effect on gut microbiota: insights for HIV-related inflammation. *AIDS Res Ther* 2020;**17:** 10.

Röhrs S, Kutzner N, Vlad A*, et al*. Chronological expression of Wnt target genes CCND1, MYC, CDKN1A, TFRC, PLF1 and Ramp3. *Cell biology international* 2009;**33:** 501-508.

Slenter DN, Kutmon M, Hanspers K*, et al*. WikiPathways: a multifaceted pathway database bridging metabolomics to other omics research. *Nucleic acids research* 2018;**46:** D661-D667.

Subramanian A, Narayan R, Corsello SM*, et al*. A next generation connectivity map: L1000 platform and the first 1,000,000 profiles. *Cell* 2017;**171:** 1437-1452. e1417.

Wang Y, An H, Liu T*, et al*. Metformin Improves Mitochondrial Respiratory Activity through Activation of AMPK. *Cell Rep* 2019;**29:** 1511-1523.e1515.

Xia X, Xia J, Yang H*, et al*. Baicalein blocked cervical carcinoma cell proliferation by targeting CCND1 via Wnt/β-catenin signaling pathway. *Artificial cells, nanomedicine, and biotechnology* 2019;**47:** 2729-2736.

Xiao Q, Werner J, Venkatachalam N*, et al*. Cross-talk between p53 and Wnt signaling in cancer. *Biomolecules* 2022;**12:** 453.

Xu C, Xu Z, Zhang Y*, et al*. β-Catenin signaling in hepatocellular carcinoma. *J Clin Invest* 2022;**132:**

Yerevanian A, Soukas AA. Metformin: Mechanisms in Human Obesity and Weight Loss. *Curr Obes Rep* 2019;**8:** 156-164.

Yevshin I, Sharipov R, Kolmykov S*, et al*. GTRD: a database on gene transcription regulation—2019 update. *Nucleic Acids Research* 2018;**47:** D100-D105.

Yi Y, Zhang W, Yi J*, et al*. Role of p53 Family Proteins in Metformin Anti-Cancer Activities. *J Cancer* 2019;**10:** 2434-2442.

Zheng L, Yang W, Wu F*, et al*. Prognostic significance of AMPK activation and therapeutic effects of metformin in hepatocellular carcinoma. *Clin Cancer Res* 2013;**19:** 5372-5380.

Zhou S, Shi X, Song C*, et al*. SNP discovery of PRKAB1 gene and their associations with growth traits in goats. *Anim Biotechnol* 2022;**33:** 1613-1619.
