# Supplementary figures and images for: Open MoA: revealing the mechanism of action (MoA) based on network topology and hierarchy
Source: Bioinformatics. 2023 Oct 31;39(11):btad666. doi: 10.1093/bioinformatics/btad666 (PMC10637856; doi:10.1093/bioinformatics/btad666)

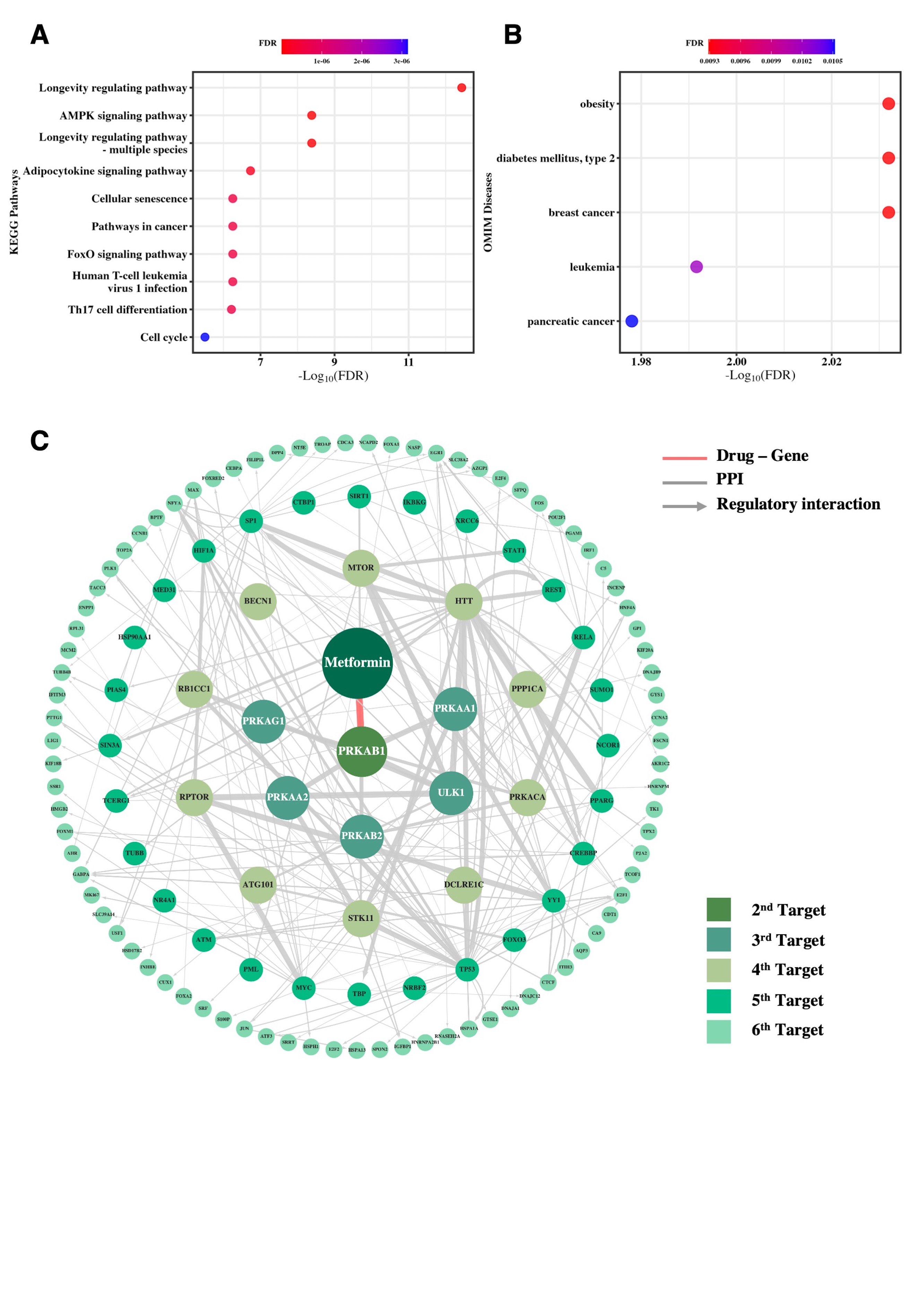

Supplement: btad666_Supplementary_Data [file btad666_supplementary_data.zip › Fig.S2.jpeg]

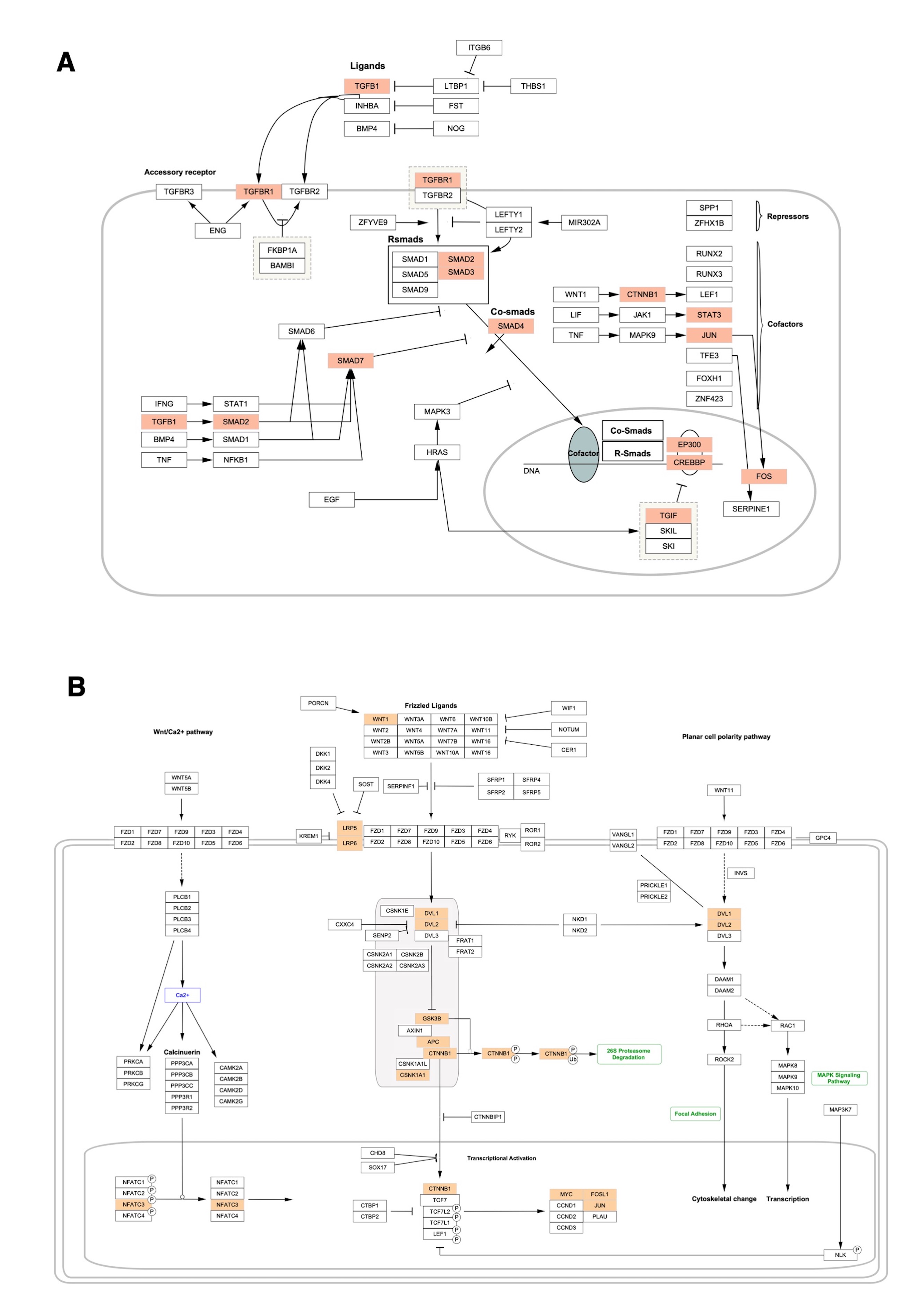

Supplement: btad666_Supplementary_Data [file btad666_supplementary_data.zip › Fig.S3.jpeg]

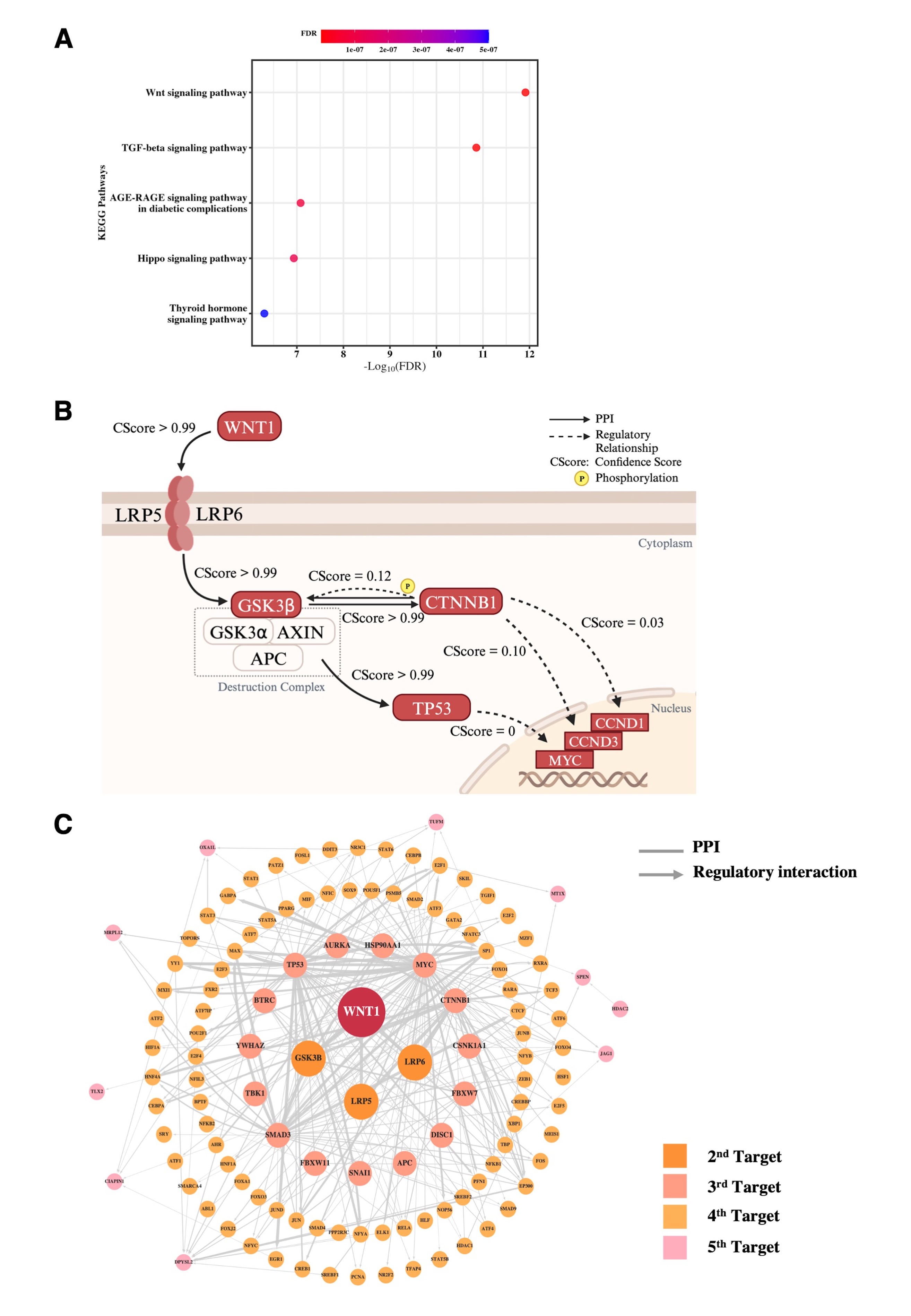

Supplement: btad666_Supplementary_Data [file btad666_supplementary_data.zip › Fig.S1.jpeg]
